# Supplementary material for: Influence of Atmospheric Conditions on Mechanical Properties of Polyamide with Different Content of Recycled Material in Selective Laser Sintering
Source: Polymers (Basel). 2022 Jun 10;14(12):2355. doi: 10.3390/polym14122355 (PMC9227374; doi:10.3390/polym14122355)
Supplement: Supplementary file 1 [file polymers-14-02355-s001.zip › polymers-1758407-supplementary.pdf]

Supplementary Materials:

Table S1. Tensile properties of original material.

No.	h, mm	b, mm	A_0, mm^2	F_m, N	R_m, MPa	ε_p, %	R_p, MPa	E, GPa
				Before exposure				
1Lxy 1	4.20	10.03	42.13	2077.7	49.32	26.50	46.17	2.026
1Lxy 2	4.18	10.02	41.88	2030.5	48.48	26.34	45.04	2.032
1Lxy 3	4.16	10.00	41.60	2038.4	49.00	35.78	45.62	2.018
$\bar{x}$	4.18	10.02	41.87	2048.9	48.93	29.54	45.61	2.025
S	0.020	0.015	0.263	25.249	0.424	5.405	0.565	0.007
1Pxy 1	4.02	10.20	41.00	2009.2	49.00	27.92	43.92	1.845
1Pxy 2	4.00	10.28	41.12	2016.9	49.05	29.58	45.19	1.964
1Pxy 3	4.01	10.22	40.98	2004.8	48.92	25.27	46.83	1.909
$\bar{x}$	4.01	10.23	41.04	2010.3	48.99	27.59	45.31	1.906
S	0.010	0.042	0.074	6.122	0.066	2.174	1.459	0.060
1Pz 1	3.98	10.02	39.88	1915.8	48.04	10.77	47.84	2.006
1Pz 2	3.98	9.99	39.76	1946.3	48.95	12.69	48.78	2.040
1Pz 3	4.07	10.09	41.07	1942.8	47.31	10.43	47.17	1.932
$\bar{x}$	4.01	10.03	40.24	1935.0	48.10	11.30	47.93	1.993
S	0.052	0.051	0.722	16.680	0.822	1.219	0.809	0.055
				Ageing in UV chamber 500 h				
1Lxy 1	4.17	9.92	41.37	2075.4	50.17	20.52	46.41	1.736
1Lxy 2	4.14	10.02	41.48	2023.5	48.78	21.68	47.26	1.857
1Lxy 3	4.17	9.96	41.53	2063.0	49.67	18.90	48.04	2.095
$\bar{x}$	4.16	9.97	41.46	2053.9	49.54	20.37	47.24	1.896
S	0.017	0.050	0.086	27.060	0.704	1.396	0.815	0.183
1Pxy 1	4.03	10.34	41.67	1993.1	47.83	24.28	43.13	1.728
1Pxy 2	4.02	10.29	41.37	1983.1	47.94	23.97	45.16	1.637
1Pxy 3	3.99	10.20	40.70	2007.6	49.33	24.15	44.80	1.831
$\bar{x}$	4.01	10.28	41.24	1994.6	48.37	24.13	44.36	1.732
S	0.021	0.071	0.497	12.348	0.836	0.156	1.083	0.097
1Pz 1	4.06	10.07	40.88	1887.2	46.16	11.85	45.83	1.599
1Pz 2	4.08	10.10	41.21	1891.9	45.91	11.13	45.55	1.665
1Pz 3	4.06	10.13	41.13	1913.3	46.52	13.08	46.27	1.754
$\bar{x}$	4.07	10.10	41.07	1897.4	46.20	12.02	45.88	1.673
S	0.012	0.030	0.169	13.895	0.307	0.986	0.363	0.078
				Ageing in UV chamber 1000 h				
1Lxy 1	4.19	10.07	42.19	1911.8	45.31	10.44	44.72	2.262
1Lxy 2	4.20	9.95	41.79	1947.8	46.61	13.58	45.77	1.695
1Lxy 3	4.20	9.96	41.83	1495.5	35.75	4.04	35.75	1.938
$\bar{x}$	4.20	9.99	41.94	1785.0	42.56	9.35	42.08	1.965
S	0.006	0.067	0.222	251.397	5.930	4.862	5.507	0.284
1Pxy 1	3.99	10.27	40.98	1856.7	45.31	12.01	44.82	1.815
1Pxy 2	3.98	10.26	40.83	1336.1	32.72	2.46	32.72	1.657
1Pxy 3	4.00	10.30	41.20	1469.6	35.67	2.88	35.67	1.777
$\bar{x}$	3.99	10.28	41.00	1554.1	37.90	5.78	37.74	1.750
S	0.010	0.021	0.184	270.382	6.585	5.396	6.309	0.082
1Pz 1	4.00	10.05	40.20	1755.5	43.67	7.70	43.50	1.849
1Pz 2	4.01	10.02	40.18	1729.8	43.05	6.85	42.93	2.183
1Pz 3	4.03	10.05	40.50	1752.1	43.26	10.83	42.82	1.953

$\bar{x}$	4.01	10.04	40.29	1745.8	43.33	8.46	43.08	1.995
S	0.015	0.017	0.180	13.995	0.315	2.095	0.365	0.171
Natural ageing								
1Lxy 1	4.16	10.05	41.81	1877.0	44.90	16.67	43.83	1.203
1Lxy 2	4.17	10.00	41.70	1832.6	43.95	16.23	43.55	1.225
1Lxy 3	4.13	10.02	41.38	1898.3	45.87	16.78	43.12	1.198
$\bar{x}$	4.15	10.02	41.63	1869.3	44.91	16.56	43.50	1.209
S	0.021	0.025	0.221	33.520	0.962	0.291	0.358	0.014
1Pxy 1	4.21	10.30	43.36	1846.0	42.57	20.15	41.06	1.162
1Pxy 2	4.16	10.27	42.72	1834.1	42.93	20.23	41.02	1.167
1Pxy 3	4.18	10.20	42.64	1854.5	43.50	20.01	41.12	1.870
$\bar{x}$	4.18	10.26	42.91	1844.9	43.00	20.13	41.07	1.400
S	0.025	0.051	0.397	10.247	0.467	0.111	0.050	0.407
1Pz 1	3.99	10.07	40.18	1727.0	42.97	10.75	42.72	1.123
1Pz 2	3.98	10.05	40.00	1720.1	43.00	10.73	42.67	1.143
1Pz 3	4.00	10.00	40.00	1723.4	43.09	10.69	42.55	1.118
$\bar{x}$	3.99	10.04	40.06	1723.5	43.02	10.72	42.65	1.128
S	0.010	0.036	0.104	3.451	0.059	0.031	0.087	0.013

Table S2. Tensile properties of original material – water absorption.

No.	h, mm	b, mm	A_0, mm^2	F_m, N	R_m, MPa	ε_p, %	R_p, MPa	E, GPa
Drying 2 h at 100 °C								
1Lxy 1	4.15	9.98	41.42	2091.1	50.49	27.39	48.10	1.696
1Lxy 2	4.20	10.00	42.00	2152.1	51.24	24.64	49.12	1.756
1Lxy 3	4.18	10.00	41.8	2127.2	50.89	24.91	47.47	1.789
$\bar{x}$	4.18	9.99	41.74	2123.5	50.87	25.65	48.23	1.747
S	0.025	0.012	0.296	30.638	0.375	1.516	0.833	0.047
1Pxy 1	4.06	10.22	41.49	2082.1	50.18	24.37	47.45	1.628
1Pxy 2	4.11	10.31	42.37	2034.8	48.02	12.23	47.65	1.725
1Pxy 3	4.05	10.21	41.35	2073.3	50.14	23.03	48.34	1.691
$\bar{x}$	4.07	10.25	41.74	2063.4	49.45	19.88	47.81	1.681
S	0.032	0.055	0.554	25.167	1.236	6.656	0.467	0.049
1Pz 1	4.09	10.08	41.23	1870.9	45.38	7.03	45.32	1.799
1Pz 2	4.05	10.07	40.78	1977.6	48.49	11.67	48.27	1.881
1Pz 3	4.03	10.14	40.86	1961.9	48.01	11.35	47.82	1.854
$\bar{x}$	4.06	10.10	40.96	1936.8	47.29	10.02	47.14	1.845
S	0.031	0.038	0.236	57.609	1.674	2.590	1.589	0.042
Water absorption 1 day								
1Lxy 1	4.17	10.00	41.70	2017.9	48.39	27.14	45.88	1.973
1Lxy 2	4.20	9.98	41.92	2012.4	48.01	28.95	44.49	2.112
1Lxy 3	4.18	10.02	41.88	1985.3	47.40	29.54	43.26	1.863
$\bar{x}$	4.18	10.00	41.83	2005.2	47.93	28.54	44.54	1.983
S	0.015	0.020	0.116	17.446	0.499	1.251	1.311	0.125
1Pxy 1	3.96	10.18	40.31	1945.9	48.27	33.48	43.30	1.806
1Pxy 2	3.94	10.30	40.58	1941.4	47.84	33.04	38.53	1.819
1Pxy 3	3.98	10.27	40.87	1967.3	48.13	28.22	42.76	1.944
$\bar{x}$	3.96	10.25	40.59	1951.5	48.08	31.58	41.53	1.856
S	0.020	0.062	0.281	13.820	0.219	2.918	2.612	0.076
1Pz 1	3.98	9.97	39.68	1879.7	47.37	13.04	47.14	1.890
1Pz 2	4.07	10.11	41.15	1895.3	46.06	16.22	45.51	1.863

1Pz 3	4.06	10.06	40.84	1777.1	43.51	7.06	43.43	1.829
$\bar{x}$	4.04	10.05	40.56	1850.7	45.65	12.11	45.36	1.861
S	0.049	0.071	0.774	64.192	1.963	4.653	1.860	0.031
Water absorption 4 days								
1Lxy 1	4.17	9.95	41.49	2028.1	48.88	27.41	44.00	1.794
1Lxy 2	4.20	10.00	42.00	1982.8	47.21	33.12	42.82	1.824
1Lxy 3	4.21	10.00	42.10	1993.0	47.34	30.28	44.24	1.970
$\bar{x}$	4.19	9.98	41.86	2001.3	47.81	30.27	43.69	1.863
S	0.021	0.029	0.326	23.756	0.929	2.855	0.760	0.094
1Pxy 1	3.99	10.38	41.42	1922.1	46.41	31.33	32.07	1.858
1Pxy 2	4.02	10.29	41.37	1926.8	46.58	31.42	41.85	1.768
1Pxy 3	4.02	10.30	41.41	1927.9	46.56	29.03	42.49	1.795
$\bar{x}$	4.01	10.32	41.40	1925.6	46.52	30.59	38.80	1.807
S	0.017	0.049	0.027	3.056	0.093	1.355	5.840	0.046
1Pz 1	4.10	10.16	41.66	1890.8	45.39	13.84	45.17	1.782
1Pz 2	4.14	10.14	41.98	1881.9	44.83	13.52	37.35	1.777
1Pz 3	4.09	10.13	41.43	1883.9	45.47	22.36	44.82	1.676
$\bar{x}$	4.11	10.14	41.69	1885.5	45.23	16.57	42.45	1.745
S	0.026	0.015	0.275	4.633	0.349	5.014	4.417	0.060
Water absorption 7 days								
1Lxy 1	4.19	10.00	41.90	1995.3	47.62	25.71	43.46	1.769
1Lxy 2	4.22	10.03	42.33	1998.7	47.22	24.00	45.15	1.638
1Lxy 3	4.26	10.04	42.77	2023.5	47.31	29.60	45.00	1.812
$\bar{x}$	4.22	10.02	42.33	2005.8	47.38	26.44	44.54	1.740
S	0.035	0.021	0.435	15.392	0.210	2.870	0.935	0.091
1Pxy 1	3.98	10.34	41.15	1921.0	46.68	29.82	39.69	1.659
1Pxy 2	3.99	10.17	40.58	1911.2	47.10	32.61	42.83	1.776
1Pxy 3	3.97	10.21	40.53	1930.2	47.62	28.40	41.37	1.964
$\bar{x}$	3.98	10.24	40.76	1920.8	47.13	30.28	41.30	1.800
S	0.010	0.089	0.346	9.490	0.471	2.142	1.571	0.154
1Pz 1	4.07	10.09	41.07	1855.8	45.19	15.10	41.80	1.720
1Pz 2	4.06	10.08	40.92	1873.9	45.79	22.51	45.38	1.743
1Pz 3	4.06	10.08	40.92	1875.2	45.82	24.01	44.94	1.975
$\bar{x}$	4.06	10.08	40.97	1868.3	45.60	20.54	44.04	1.813
S	0.006	0.006	0.082	10.857	0.355	4.771	1.952	0.141
Water absorption 14 days								
1Lxy 1	4.18	10.06	42.05	1976.0	46.99	28.51	44.18	1.776
1Lxy 2	4.18	10.01	41.84	2017.6	48.22	23.98	45.59	1.604
1Lxy 3	4.19	10.06	42.15	1986.2	47.12	29.62	44.43	1.555
$\bar{x}$	4.18	10.04	42.01	1993.3	47.44	27.37	44.73	1.645
S	0.006	0.029	0.158	21.705	0.676	2.988	0.752	0.116
1Pxy 1	3.99	10.28	41.02	1916.3	46.72	29.31	42.94	1.695
1Pxy 2	4.01	10.29	41.26	1906.3	46.20	33.08	41.35	1.706
1Pxy 3	4.00	10.27	41.08	1946.8	47.39	28.52	41.83	1.819
$\bar{x}$	4.00	10.28	41.12	1923.2	46.77	30.30	42.04	1.740
S	0.010	0.010	0.128	21.064	0.597	2.437	0.816	0.069
1Pz 1	4.09	10.10	41.31	1843.2	44.62	11.43	44.38	1.439
1Pz 2	4.08	10.07	41.09	1848.0	44.98	17.54	44.35	1.615
1Pz 3	4.06	10.10	41.01	1791.6	43.69	11.21	43.34	1.594
$\bar{x}$	4.08	10.09	41.13	1827.6	44.43	13.39	44.02	1.549
S	0.015	0.017	0.157	31.308	0.666	3.593	0.592	0.096
Water absorption 28 days								

1Lxy 1	4.18	10.08	42.13	1896.5	45.01	31.11	41.88	1.507
1Lxy 2	4.19	10.02	41.98	1888.4	44.98	36.60	40.10	1.544
1Lxy 3	4.20	10.06	42.25	1872.6	44.32	33.19	39.82	1.488
$\bar{x}$	4.19	10.05	42.12	1885.8	44.77	33.63	40.6	1.513
S	0.010	0.031	0.134	12.140	0.390	2.772	1.117	0.028
1Pxy 1	3.96	10.35	40.99	1807.5	44.10	34.66	36.77	1.385
1Pxy 2	4.02	10.33	41.53	1844.6	44.42	34.55	40.25	1.419
1Pxy 3	4.00	10.31	41.24	1805.1	43.77	36.59	38.48	1.328
$\bar{x}$	3.99	10.33	41.25	1819.1	44.10	35.27	38.50	1.377
S	0.031	0.020	0.270	22.164	0.325	1.147	1.740	0.046
1Pz 1	4.12	10.12	41.69	1742.4	41.79	11.77	41.65	1.391
1Pz 2	4.11	10.16	41.76	1728.8	41.40	11.90	41.08	1.369
1Pz 3	4.05	10.1	40.91	1693.9	41.41	10.56	40.86	1.263
$\bar{x}$	4.09	10.13	41.45	1721.7	41.53	11.41	41.20	1.341
S	0.038	0.031	0.475	25.029	0.222	0.739	0.408	0.068

Table S3. Tensile properties of mixed material.

No.	h, mm	b, mm	A_0, mm^2	F_m, N	R_m, MPa	ε_p, %	R_p, MPa	E, GPa
Before exposure								
M1Lxy 1	4.17	10.09	42.08	2033	48.32	24.08	44.71	1.724
M1Lxy 2	4.13	9.99	41.26	1996	48.38	25.05	43.23	2.057
M1Lxy 3	4.15	9.96	41.33	1994	48.24	24.67	44.37	1.761
$\bar{x}$	4.15	10.01	41.56	2007.7	48.31	24.60	44.10	1.847
S	0.020	0.068	0.451	21.962	0.070	0.489	0.775	0.183
M1Pxy 1	4.07	10.32	42.00	1985	47.25	19.51	40.43	1.646
M1Pxy 2	4.11	10.27	42.21	1992	47.18	25.19	40.73	2.435
M1Pxy 3	4.07	10.24	41.68	1943	46.62	24.19	40.69	1.834
$\bar{x}$	4.08	10.28	41.96	1973.3	47.02	22.96	40.62	1.972
S	0.023	0.040	0.269	26.502	0.345	3.032	0.163	0.412
M1Pz 1	4.01	10.01	40.14	1572	39.16	6.09	37.62	1.694
M1Pz 2	4.00	10.02	40.08	1683	42.00	6.17	41.89	2.000
M1Pz 3	4.00	10.00	40.00	1426	35.65	5.04	28.17	1.739
$\bar{x}$	4.00	10.01	40.07	1560.3	38.94	5.76	35.89	1.811
S	0.006	0.010	0.070	128.897	3.181	0.632	7.021	0.165
Ageing in UV chamber 1000 h								
M1Lxy 1	4.16	9.99	41.56	1995	48.00	19.02	46.10	1.241
M1Lxy 2	4.18	10.08	42.13	2010	47.72	17.12	45.95	1.260
M1Lxy 3	4.14	10.00	41.40	1997	48.24	17.51	46.88	1.319
$\bar{x}$	4.16	10.02	41.70	2000.7	47.99	17.88	46.31	1.273
S	0.020	0.049	0.386	8.145	0.260	1.004	0.499	0.041
M1Pxy 1	4.09	10.26	41.96	1960	46.70	17.30	44.96	1.267
M1Pxy 2	4.02	10.24	41.16	1951	47.39	17.76	44.82	1.249
M1Pxy 3	4.13	10.27	42.42	2006	47.29	18.75	44.72	1.256
$\bar{x}$	4.08	10.26	41.85	1972.3	47.13	17.94	44.83	1.257
S	0.056	0.015	0.633	29.501	0.373	0.741	0.121	0.009
M1Pz 1	3.98	9.97	39.68	1418	35.73	4.60	35.65	1.208
M1Pz 2	3.98	10.00	39.80	1690	42.46	7.55	42.15	1.283
M1Pz 3	4.01	10.01	40.14	1274	31.74	3.76	31.74	1.183
$\bar{x}$	3.99	9.99	39.87	1460.7	36.64	5.30	36.51	1.225
S	0.017	0.021	0.238	211.257	5.418	1.994	5.258	0.052

Natural ageing								
M1Lxy 1	4.16	9.97	41.48	1898	45.75	16.74	43.82	1.225
M1Lxy 2	4.11	10.00	41.10	1892	46.03	16.55	43.01	1.234
M1Lxy 3	4.09	9.98	40.82	1901	46.57	17.01	43.76	1.201
$\bar{x}$	4.12	9.98	41.13	1897.0	46.12	16.77	43.53	1.220
S	0.036	0.015	0.330	4.583	0.418	0.231	0.451	0.017
M1Pxy 1	4.07	10.21	41.55	1909	45.93	19.59	42.47	1.282
M1Pxy 2	4.01	10.19	40.86	1889	46.23	19.43	42.43	1.279
M1Pxy 3	4.05	10.10	40.91	1905	46.57	19.78	42.53	1.305
$\bar{x}$	4.04	10.17	41.11	1901.0	46.24	19.60	42.48	1.289
S	0.031	0.059	0.388	10.583	0.321	0.175	0.050	0.014
M1Pz 1	4.01	10.00	40.10	1340	33.41	4.10	33.38	1.113
M1Pz 2	4.02	10.01	40.24	1332	33.10	4.01	33.23	1.231
M1Pz 3	4.00	10.01	40.04	1345	33.59	4.23	33.34	1.110
$\bar{x}$	4.01	10.01	40.13	1339.0	33.37	4.11	33.32	1.151
S	0.010	0.006	0.103	6.557	0.248	0.110	0.078	0.069
Water absorption 28 days								
M1Lxy 1	4.16	10.00	41.60	1907	45.84	23.51	35.68	1.849
M1Lxy 2	4.16	9.96	41.43	1909	46.08	24.08	40.87	1.816
M1Lxy 3	4.16	9.98	41.52	1904	45.85	26.77	37.70	1.503
$\bar{x}$	4.16	9.98	41.52	1906.7	45.92	24.79	38.08	1.723
S	0.000	0.020	0.083	2.517	0.136	1.741	2.616	0.191
M1Pxy 1	4.04	10.25	41.41	1886	45.53	23.20	24.08	1.7810
M1Pxy 2	4.07	10.24	41.68	1896	45.48	25.00	39.52	1.5870
M1Pxy 3	4.04	10.25	41.41	1863	44.99	24.95	40.40	1.6010
$\bar{x}$	4.05	10.25	41.50	1881.7	45.33	24.38	34.67	1.656
S	0.017	0.006	0.154	16.921	0.298	1.025	9.179	0.108
M1Pz 1	4.02	10.00	40.20	1662	41.34	10.05	41.09	1.492
M1Pz 2	4.01	9.98	40.02	1703	42.56	12.21	41.89	1.631
M1Pz 3	4.01	10.04	40.26	1517	37.67	7.16	37.45	1.565
$\bar{x}$	4.01	10.01	40.16	1627.3	40.52	9.81	40.14	1.563
S	0.006	0.031	0.125	97.726	2.545	2.532	2.367	0.070

Table S4. Tensile properties of 100% recycled material.

No.	h, mm	b, mm	A_0, mm²	F_m, N	R_m, MPa	ε_p, %	R_p, MPa	E, GPa
Before exposure								
R1Lxy 1	4.15	9.8	40.67	1867	45.90	30.31	38.29	1.525
R1Lxy 2	4.17	10.06	41.95	1938	46.19	23.25	38.33	1.605
R1Lxy 3	4.17	10.00	41.70	1915	45.92	20.63	38.00	1.607
$\bar{x}$	4.16	9.95	41.44	1906.7	46.00	24.73	38.21	1.579
S	0.012	0.136	0.679	36.226	0.162	5.007	0.180	0.047
R1Pxy 1	3.99	10.18	40.62	1816	44.71	25.32	27.24	1.611
R1Pxy 2	4.05	10.25	41.51	1841	44.34	19.10	37.57	1.631
R1Pxy 3	3.98	10.23	40.72	1795	44.08	20.88	37.81	1.868
$\bar{x}$	4.01	10.22	40.95	1817.3	44.38	21.77	34.21	1.703
S	0.038	0.036	0.491	23.029	0.317	3.203	6.035	0.143
R1Pz 1	4.08	10.01	40.84	1920	47.02	10.42	38.65	2.099
R1Pz 2	4.02	10.01	40.24	1901	47.25	10.19	29.06	1.823
R1Pz 3	4.01	10.00	40.10	1857	46.32	10.27	44.78	1.728
$\bar{x}$	4.04	10.01	40.39	1892.7	46.86	10.29	37.50	1.883

S	0.038	0.006	0.394	32.316	0.484	0.117	7.923	0.193
Ageing in UV chamber 1000 h								
R1Lxy 1	4.15	9.85	40.88	1817	44.45	22.47	41.89	1.192
R1Lxy 2	4.13	9.89	40.85	1810	44.32	19.74	41.07	1.168
R1Lxy 3	4.14	9.99	41.36	1804	43.61	24.89	39.37	1.203
$\bar{x}$	4.14	9.91	41.03	1810.3	44.13	22.37	40.78	1.188
S	0.010	0.072	0.287	6.506	0.452	2.577	1.285	0.018
R1Pxy 1	4.03	10.21	41.15	1728	42.00	22.99	39.41	1.181
R1Pxy 2	4.02	10.2	41.00	1721	41.98	22.41	39.84	1.159
R1Pxy 3	3.99	10.21	40.74	1718	42.17	23.85	38.06	1.161
$\bar{x}$	4.01	10.21	40.96	1722.3	42.05	23.08	39.10	1.167
S	0.021	0.006	0.207	5.132	0.104	0.725	0.929	0.012
R1Pz 1	4.08	10.05	41.00	1741	42.45	10.13	41.87	1.211
R1Pz 2	4.07	10.03	40.82	1739	42.61	11.21	42.14	1.209
R1Pz 3	4.08	10.04	40.96	1739	42.46	10.13	41.83	1.207
$\bar{x}$	4.08	10.04	40.93	1739.7	42.51	10.49	41.95	1.209
S	0.006	0.010	0.095	1.155	0.090	0.624	0.169	0.002
Natural ageing								
R1Lxy 1	4.09	9.96	40.74	1788	43.90	16.68	41.72	1.238
R1Lxy 2	4.05	9.98	40.42	1775	43.91	16.21	41.73	1.204
R1Lxy 3	4.04	10.01	40.44	1952	48.27	16.71	41.54	1.245
$\bar{x}$	4.06	9.98	40.53	1838.3	45.36	16.53	41.66	1.229
S	0.026	0.025	0.177	98.653	2.518	0.280	0.107	0.022
R1Pxy 1	3.93	10.21	40.13	1683	41.93	13.92	40.14	1.157
R1Pxy 2	3.98	10.11	40.24	1657	41.18	14.02	39.98	1.145
R1Pxy 3	3.96	10.19	40.35	1698	42.08	13.89	40.32	1.160
$\bar{x}$	3.96	10.17	40.24	1679.3	41.73	13.94	40.15	1.154
S	0.025	0.053	0.114	20.744	0.482	0.068	0.170	0.008
R1Pz 1	4.02	9.99	40.16	1583	39.41	8.15	38.90	1.155
R1Pz 2	4.01	10.00	40.10	1580	39.40	8.23	38.62	1.161
R1Pz 3	3.99	10.02	39.98	1556	38.92	7.98	39.14	1.152
$\bar{x}$	4.01	10.00	40.08	1573.0	39.24	8.12	38.89	1.156
S	0.015	0.015	0.092	14.799	0.281	0.127	0.260	0.005
Water absorption 28 days								
R1Lxy 1	4.19	9.90	41.48	1737	41.87	36.21	28.91	1.105
R1Lxy 2	4.14	9.95	41.19	1724	41.86	37.73	33.59	1.090
R1Lxy 3	4.28	9.85	42.16	1690	40.08	25.44	33.68	1.050
$\bar{x}$	4.20	9.90	41.61	1717.0	41.27	33.13	32.06	1.082
S	0.071	0.050	0.495	24.269	1.031	6.700	2.728	0.028
R1Pxy 1	4.03	10.19	41.07	1658	40.38	39.99	32.33	1.037
R1Pxy 2	4.04	10.29	41.57	1661	39.97	32.02	33.34	1.046
R1Pxy 3	4.05	10.32	41.80	1658	39.67	29.28	32.22	1.011
$\bar{x}$	4.04	10.27	41.48	1659.0	40.01	33.76	32.63	1.031
S	0.010	0.068	0.374	1.732	0.356	5.564	0.617	0.018
R1Pz 1	3.97	10.00	39.70	1379	34.74	8.25	33.81	1.081
R1Pz 2	4.04	10.03	40.52	1632	40.28	11.95	39.36	1.160
R1Pz 3	4.06	10.02	40.68	1576	38.74	11.08	25.23	1.121
$\bar{x}$	4.02	10.02	40.30	1529.0	37.92	10.43	32.80	1.121
S	0.047	0.015	0.526	132.887	2.860	1.933	7.119	0.040

Table S5. Flexural properties of original material.

No.	h, mm	b, mm	A_0, mm²	F_{max}, N	σ_{fm}, MPa	ε_{fm}, %	S_{max}, mm	ε_{fp}, %	σ_{fp}, MPa	E_f, GPa
					Before exposure					
2Lxy 1	4.20	9.97	41.87	120.3	65.69	7.56	12.29	9.98	63.24	1.584
2Lxy 2	4.17	10.01	41.74	116.9	64.50	7.54	12.34	15.90	41.56	1.504
2Lxy 3	4.15	10.02	41.58	116.9	65.06	6.96	11.45	9.89	63.20	1.493
$\bar{x}$	4.17	10.00	41.73	118.0	65.08	7.35	12.03	11.92	56.00	1.527
S	0.025	0.026	0.146	1.963	0.595	0.341	0.500	3.444	12.505	0.050
2Pxy 1	3.97	10.28	40.81	105.7	62.63	7.05	12.13	9.43	59.96	1.541
2Pxy 2	4.00	10.25	41.00	110.2	64.51	7.64	13.05	9.87	61.23	1.690
2Pxy 3	4.01	10.25	41.10	107.9	62.88	8.12	13.83	9.99	58.94	1.567
$\bar{x}$	3.99	10.26	40.97	107.9	63.34	7.61	13.00	9.76	60.04	1.599
S	0.021	0.017	0.148	2.250	1.021	0.535	0.851	0.294	1.147	0.080
2Pz 1	4.00	9.98	39.92	104.6	62.89	7.44	12.69	9.93	56.25	1.629
2Pz 2	4.02	10.00	40.20	103.4	61.45	7.15	12.14	10.40	56.01	1.569
2Pz 3	4.05	10.04	40.66	105.7	61.62	7.31	12.32	14.63	47.19	1.619
$\bar{x}$	4.02	10.01	40.26	104.6	61.99	7.30	12.38	11.65	53.15	1.606
S	0.025	0.031	0.375	1.150	0.787	0.144	0.280	2.587	5.163	0.032
					Ageing in UV chamber 500 h					
2Lxy 1	4.17	10.03	41.83	119.3	65.64	7.98	13.07	10.27	63.16	1.602
2Lxy 2	4.21	9.96	41.93	121.5	66.07	7.58	12.29	10.02	63.00	1.493
2Lxy 3	4.16	10.03	41.72	120.3	66.56	7.17	11.77	9.96	63.47	1.497
$\bar{x}$	4.18	10.01	41.83	120.4	66.09	7.58	12.38	10.08	63.21	1.531
S	0.026	0.040	0.103	1.102	0.460	0.407	0.654	0.165	0.239	0.062
2Pxy 1	4.03	10.27	41.39	108.0	62.16	7.44	12.61	9.57	59.57	1.493
2Pxy 2	3.95	10.29	40.65	108.0	64.58	7.46	12.90	10.35	59.85	1.665
2Pxy 3	4.06	10.24	41.57	111.3	63.33	8.37	14.07	9.69	60.77	1.459
$\bar{x}$	4.01	10.27	41.20	109.1	63.36	7.76	13.19	9.87	60.06	1.539
S	0.057	0.025	0.491	1.905	1.210	0.528	0.773	0.419	0.628	0.110
2Pz 1	4.07	10.06	40.94	110.3	63.51	7.20	12.08	11.61	56.37	1.483
2Pz 2	4.02	10.09	40.56	106.8	62.91	6.93	11.77	11.01	56.25	1.549
2Pz 3	4.04	10.04	40.56	105.8	61.95	7.10	12.00	9.98	56.06	1.535
$\bar{x}$	4.04	10.06	40.69	107.6	62.79	7.08	11.95	10.87	56.23	1.522
S	0.025	0.025	0.221	2.363	0.787	0.137	0.161	0.825	0.156	0.035
					Ageing in UV chamber 1000 h					
2Lxy 1	4.17	9.96	41.53	137.3	74.52	8.24	13.35	13.20	64.75	1.612
2Lxy 2	4.22	9.96	42.03	138.4	73.58	7.92	12.73	12.79	66.41	1.606
2Lxy 3	4.15	10.05	41.71	136.2	74.03	7.38	12.02	13.71	61.80	1.749
$\bar{x}$	4.18	9.99	41.76	137.3	74.04	7.85	12.70	13.23	64.32	1.656
S	0.036	0.052	0.253	1.100	0.470	0.436	0.666	0.461	2.335	0.081
2Pxy 1	3.97	10.31	40.93	126.1	74.47	7.42	12.76	12.45	65.84	1.832
2Pxy 2	4.01	10.28	41.22	126.1	73.20	7.25	12.33	12.60	64.06	1.719
2Pxy 3	3.99	10.27	40.98	127.2	74.69	7.95	13.60	12.70	64.76	1.828
$\bar{x}$	3.99	10.29	41.04	126.5	74.12	7.54	12.90	12.58	64.89	1.793
S	0.020	0.021	0.157	0.635	0.804	0.367	0.646	0.126	0.897	0.064
2Pz 1	4.05	10.04	40.66	122.7	71.53	7.33	12.36	10.07	64.01	1.810
2Pz 2	4.02	10.04	40.36	121.6	71.92	7.15	12.14	7.79	71.92	1.636
2Pz 3	4.04	10.02	40.48	117.1	68.71	5.99	10.12	8.96	67.50	1.547
$\bar{x}$	4.04	10.03	40.50	120.5	70.72	6.82	11.54	8.94	67.81	1.664
S	0.015	0.012	0.152	2.967	1.752	0.731	1.235	1.140	3.964	0.134

					Natural ageing					
2Lxy 1	4.22	10.00	42.20	133.9	72.15	8.03	13.00	13.01	61.86	1.626
2Lxy 2	4.16	10.02	41.68	123.1	71.98	7.98	13.50	13.14	61.40	1.601
2Lxy 3	4.18	10.01	41.84	135.4	73.15	8.12	12.40	12.87	62.55	1.636
$\bar{x}$	4.19	10.01	41.91	130.8	72.43	8.04	12.97	13.01	61.94	1.621
S	0.031	0.010	0.265	6.710	0.632	0.071	0.551	0.135	0.579	0.018
2Pxy 1	4.03	10.29	41.47	119.3	68.50	7.19	12.17	12.78	58.79	0.935
2Pxy 2	4.00	10.24	40.96	120.1	68.01	7.01	12.54	12.77	57.89	0.968
2Pxy 3	4.02	10.20	41.00	118.5	69.12	7.34	12.21	12.04	58.56	0.924
$\bar{x}$	4.02	10.24	41.14	119.3	68.54	7.18	12.31	12.53	58.41	0.942
S	0.015	0.045	0.282	0.820	0.556	0.165	0.203	0.424	0.468	0.023
2Pz 1	4.03	10.05	40.50	115.8	68.14	6.97	11.80	12.18	52.50	1.382
2Pz 2	3.97	10.00	39.70	115.1	68.01	6.51	11.54	12.01	52.87	1.300
2Pz 3	4.02	10.09	40.56	116.2	68.81	6.35	11.33	12.23	52.01	1.412
$\bar{x}$	4.01	10.05	40.25	115.7	68.32	6.61	11.56	12.14	52.46	1.365
S	0.032	0.045	0.481	0.557	0.429	0.320	0.235	0.115	0.431	0.058

Table S6. Flexural properties of original material – water absorption.

No.	h, mm	b, mm	A_0, mm^2	F_{max}, N	σ_{fm}, MPa	ε_{fm}, %	S_{max}, mm	ε_{fp}, %	σ_{fp}, MPa	E_f, GPa
					Drying 6 h at 100 °C					
2Lxy 1	4.18	9.98	41.72	119.2	65.62	7.56	12.35	10.40	63.15	1.538
2Lxy 2	4.19	9.97	41.77	122.6	67.24	7.88	12.84	10.47	63.54	1.540
2Lxy 3	4.20	9.98	41.92	121.4	66.23	7.49	12.17	9.95	64.40	1.615
$\bar{x}$	4.19	9.98	41.80	121.1	66.36	7.64	12.45	10.27	63.70	1.564
S	0.010	0.006	0.103	1.724	0.818	0.208	0.347	0.284	0.640	0.044
2Pxy 1	4.03	10.25	41.31	110.2	63.55	6.66	11.28	10.09	60.32	1.594
2Pxy 2	3.98	10.27	40.87	112.4	66.36	7.15	12.26	9.53	63.70	1.745
2Pxy 3	4.02	10.26	41.25	115.8	67.08	7.61	12.93	9.54	64.47	1.706
$\bar{x}$	4.01	10.26	41.14	112.8	65.66	7.14	12.16	9.72	62.83	1.682
S	0.026	0.010	0.234	2.821	1.865	0.476	0.830	0.322	2.208	0.078
2Pz 1	3.99	10.03	40.02	111.3	66.94	7.12	12.18	9.46	64.24	1.682
2Pz 2	4.06	10.00	40.60	112.4	65.49	7.99	13.43	9.73	62.87	1.621
2Pz 3	4.01	10.04	40.26	112.4	66.87	6.97	11.87	9.59	64.87	1.731
$\bar{x}$	4.02	10.02	40.29	112.0	66.43	7.36	12.49	9.60	63.99	1.678
S	0.036	0.021	0.292	0.635	0.818	0.549	0.826	0.135	1.023	0.055
					Water absorption 1 day					
2Lxy 1	4.12	9.95	40.99	116.9	66.47	6.67	11.25	9.66	63.92	1.833
2Lxy 2	4.13	10.02	41.38	111.3	62.55	7.12	11.76	9.67	60.02	1.696
2Lxy 3	4.21	10.13	42.65	112.4	60.13	7.66	12.42	11.81	53.52	1.466
$\bar{x}$	4.15	10.03	41.67	113.5	63.05	7.15	11.81	10.38	59.15	1.665
S	0.049	0.091	0.864	2.967	3.199	0.497	0.587	1.236	5.254	0.185
2Pxy 1	3.97	10.22	40.57	102.3	61.00	6.72	11.56	9.37	59.66	1.638
2Pxy 2	3.99	10.29	41.06	101.2	59.30	7.17	12.26	9.42	56.67	1.551
2Pxy 3	3.99	10.22	40.78	103.4	61.04	6.88	11.77	9.51	58.38	1.528
$\bar{x}$	3.98	10.24	40.80	102.3	60.45	6.92	11.86	9.43	58.24	1.572
S	0.012	0.040	0.243	1.100	0.993	0.225	0.359	0.072	1.500	0.058
2Pz 1	4.00	9.99	39.96	102.3	61.47	6.78	11.57	9.50	59.43	1.558
2Pz 2	3.99	9.99	39.86	101.2	61.09	6.60	11.29	9.65	59.06	1.632
2Pz 3	4.02	9.98	40.12	102.3	60.92	7.46	12.66	10.29	56.90	1.417

$\bar{x}$	4.00	9.99	39.98	101.9	61.16	6.95	11.84	9.81	58.46	1.536
S	0.015	0.006	0.131	0.635	0.282	0.452	0.724	0.418	1.366	0.109
Water absorption 4 days										
2Lxy 1	4.19	10.05	42.11	114.7	62.41	7.75	12.63	9.90	60.59	1.473
2Lxy 2	4.20	10.09	42.38	112.4	60.65	8.42	13.68	9.99	58.22	1.450
2Lxy 3	4.23	10.14	42.89	116.9	62.53	7.43	12.04	10.67	59.54	1.512
$\bar{x}$	4.21	10.09	42.46	114.7	61.86	7.86	12.78	10.19	59.45	1.478
S	0.021	0.045	0.398	2.250	1.052	0.505	0.831	0.421	1.188	0.031
2Pxy 1	4.01	10.26	41.14	102.3	59.56	7.34	12.49	9.53	56.94	1.413
2Pxy 2	4.01	10.27	41.18	100.1	58.19	7.00	11.92	9.66	56.21	1.362
2Pxy 3	4.00	10.28	41.12	103.4	60.38	7.54	12.86	9.46	58.42	1.426
$\bar{x}$	4.01	10.27	41.15	101.9	59.38	7.29	12.42	9.55	57.19	1.400
S	0.006	0.010	0.032	1.680	1.106	0.272	0.474	0.105	1.126	0.034
2Pz 1	4.04	10.04	40.56	102.3	59.96	6.94	11.72	9.60	57.97	1.523
2Pz 2	4.07	10.07	40.98	103.4	59.54	7.06	11.84	11.41	53.72	1.518
2Pz 3	4.06	10.07	40.88	102.3	59.19	7.68	12.92	9.60	56.59	1.493
$\bar{x}$	4.06	10.06	40.81	102.7	59.56	7.23	12.16	10.20	56.09	1.511
S	0.015	0.017	0.221	0.635	0.386	0.401	0.661	1.048	2.168	0.016
Water absorption 7 days										
2Lxy 1	4.19	10.04	42.07	114.7	62.47	7.52	12.25	10.07	60.02	1.543
2Lxy 2	4.09	10.03	41.02	107.9	61.77	7.59	12.67	10.02	58.56	1.369
2Lxy 3	4.17	10.02	41.78	114.7	63.20	7.46	12.21	9.86	61.35	1.433
$\bar{x}$	4.15	10.03	41.62	112.4	62.48	7.52	12.38	9.98	59.98	1.448
S	0.053	0.010	0.540	3.926	0.715	0.065	0.255	0.110	1.396	0.088
2Pxy 1	3.98	10.25	40.80	100.1	59.19	7.39	12.67	9.44	57.86	1.474
2Pxy 2	3.97	10.28	40.81	102.3	60.64	7.03	12.09	9.47	58.63	1.516
2Pxy 3	3.98	10.25	40.80	100.1	59.19	7.36	12.62	10.13	55.84	1.452
$\bar{x}$	3.98	10.26	40.80	100.8	59.67	7.26	12.46	9.68	57.44	1.481
S	0.006	0.017	0.010	1.270	0.837	0.199	0.321	0.392	1.441	0.033
2Pz 1	3.99	10.07	40.18	102.3	61.29	7.50	12.82	9.65	59.25	1.536
2Pz 2	4.00	10.03	40.12	102.3	61.23	7.36	12.56	10.03	58.53	1.645
2Pz 3	4.05	10.03	40.62	100.1	58.41	6.81	11.47	9.58	56.43	1.500
$\bar{x}$	4.01	10.04	40.31	101.6	60.31	7.22	12.28	9.75	58.07	1.560
S	0.032	0.023	0.274	1.270	1.646	0.366	0.716	0.242	1.465	0.076
Water absorption 14 days										
2Lxy 1	4.24	9.99	42.36	109.1	58.29	7.53	12.12	10.87	54.09	1.545
2Lxy 2	4.19	10.07	42.19	110.2	59.84	7.48	12.19	10.76	56.80	1.265
2Lxy 3	4.13	10.06	41.55	104.6	58.52	7.49	12.39	10.25	56.00	1.333
$\bar{x}$	4.19	10.04	42.03	108.0	58.88	7.50	12.23	10.63	55.63	1.381
S	0.055	0.044	0.428	2.967	0.836	0.024	0.140	0.331	1.392	0.146
2Pxy 1	4.00	10.29	41.16	95.6	55.74	7.33	12.51	10.13	52.45	1.424
2Pxy 2	3.96	10.26	40.63	94.4	56.36	7.16	12.34	10.10	52.33	1.392
2Pxy 3	4.00	10.23	40.92	93.3	54.75	7.21	12.30	9.94	52.11	1.383
$\bar{x}$	3.99	10.26	40.90	94.5	55.62	7.23	12.38	10.06	52.30	1.400
S	0.023	0.030	0.266	1.125	0.812	0.089	0.112	0.103	0.172	0.022
2Pz 1	4.08	10.11	41.25	96.7	55.16	7.45	12.47	10.32	51.97	1.348
2Pz 2	4.05	10.05	40.70	93.3	54.36	7.49	12.63	10.11	51.74	1.391
2Pz 3	4.07	10.07	40.98	96.7	55.65	7.82	13.12	10.21	52.43	1.453
$\bar{x}$	4.07	10.08	40.98	95.6	55.06	7.59	12.74	10.21	52.05	1.397
S	0.015	0.031	0.273	1.934	0.651	0.203	0.339	0.105	0.351	0.053
Water absorption 28 days										
2Lxy 1	4.21	10.04	42.27	104.6	56.43	7.54	12.22	10.50	54.00	1.238

2Lxy 2	4.23	10.01	42.34	101.3	54.27	8.29	13.38	12.76	46.42	1.169
2Lxy 3	4.22	10.06	42.45	103.5	55.46	7.80	12.62	10.33	53.05	1.336
$\bar{x}$	4.22	10.04	42.35	103.1	55.39	7.88	12.74	11.20	51.16	1.248
S	0.010	0.025	0.093	1.680	1.082	0.383	0.589	1.357	4.129	0.084
2Pxy 1	4.02	10.26	41.25	94.5	54.71	7.37	12.52	9.55	52.75	1.291
2Pxy 2	3.98	10.25	40.80	90.0	53.21	7.40	12.69	9.52	50.55	1.226
2Pxy 3	4.00	10.27	41.08	87.8	51.27	8.20	14.00	9.61	49.28	1.203
$\bar{x}$	4.00	10.26	41.04	90.8	53.06	7.66	13.07	9.56	50.86	1.240
S	0.020	0.010	0.228	3.437	1.725	0.471	0.810	0.048	1.756	0.046
2Pz 1	4.07	10.09	41.07	93.3	53.62	8.14	13.65	10.84	49.74	1.282
2Pz 2	4.06	10.06	40.84	87.8	50.80	7.28	12.23	9.63	48.20	1.309
2Pz 3	4.02	10.04	40.36	88.8	52.57	7.51	12.76	10.56	48.58	1.222
$\bar{x}$	4.05	10.06	40.76	90.0	52.33	7.64	12.88	10.34	48.84	1.271
S	0.026	0.025	0.361	2.967	1.425	0.447	0.718	0.631	0.802	0.045

Table S7. Flexural properties of mixed material.

No.	h, mm	b, mm	A_0, mm^2	F_{max}, N	σ_{fm}, MPa	ε_{fm}, %	S_{max}, mm	ε_{fp}, %	σ_{fp}, MPa	E_f, GPa
					Before exposure					
M2Lxy 1	4.16	10.05	41.81	127.1	70.16	7.38	12.11	12.87	60.83	1.463
M2Lxy 2	4.17	10.10	42.12	128.2	70.08	8.05	13.19	13.13	62.09	1.300
M2Lxy 3	4.14	10.01	41.44	124.8	69.86	7.62	12.57	13.89	59.79	1.538
$\bar{x}$	4.17	10.00	41.73	118.0	65.08	7.35	12.03	11.92	56.00	1.527
S	0.025	0.026	0.146	1.963	0.595	0.341	0.500	3.444	12.505	0.050
M2Pxy 1	3.97	10.28	40.81	105.7	62.63	7.05	12.13	9.43	59.96	1.541
M2Pxy 2	4.00	10.25	41.00	110.2	64.51	7.64	13.05	9.87	61.23	1.690
M2Pxy 3	4.01	10.25	41.10	107.9	62.88	8.12	13.83	9.99	58.94	1.567
$\bar{x}$	3.99	10.26	40.97	107.9	63.34	7.61	13.00	9.76	60.04	1.599
S	0.021	0.017	0.148	2.250	1.021	0.535	0.851	0.294	1.147	0.080
M2Pz 1	4.00	9.98	39.92	104.6	62.89	7.44	12.69	9.93	56.25	1.629
M2Pz 2	4.02	10.00	40.20	103.4	61.45	7.15	12.14	10.40	56.01	1.569
M2Pz 3	4.05	10.04	40.66	105.7	61.62	7.31	12.32	14.63	47.19	1.619
$\bar{x}$	4.02	10.01	40.26	104.6	61.99	7.30	12.38	11.65	53.15	1.606
S	0.025	0.031	0.375	1.150	0.787	0.144	0.280	2.587	5.163	0.032
					Ageing in UV chamber 1000 h					
M2Lxy 1	4.16	10.07	41.89	75.3	41.51	2.44	4.01	2.71	41.25	1.629
M2Lxy 2	4.17	10.15	42.33	129.3	70.33	7.21	11.80	12.14	62.39	1.639
M2Lxy 3	4.15	10.05	41.71	131.6	72.96	7.59	12.49	12.16	66.72	1.696
$\bar{x}$	4.16	10.09	41.97	112.1	61.60	5.75	9.43	9.00	56.79	1.655
S	0.010	0.053	0.317	31.833	17.448	2.867	4.708	5.449	13.628	0.036
M2Pxy 1	4.00	10.15	40.60	125.9	74.45	7.61	12.98	12.16	65.79	1.689
M2Pxy 2	3.99	10.12	40.38	119.3	71.06	7.15	12.23	13.12	58.30	1.720
M2Pxy 3	4.04	10.16	41.05	129.4	74.88	7.07	11.94	13.13	63.83	1.705
$\bar{x}$	4.01	10.14	40.68	124.9	73.46	7.27	12.38	12.80	62.64	1.705
S	0.026	0.021	0.340	5.129	2.092	0.290	0.537	0.557	3.884	0.016
M2Pz 1	4.05	10.08	40.82	106.8	62.04	5.58	9.40	7.14	60.00	1.672
M2Pz 2	4.07	10.08	41.03	118.1	67.90	7.30	12.24	8.50	63.75	1.674
M2Pz 3	4.00	9.99	39.96	117.0	70.27	7.08	12.09	9.52	59.98	1.682
$\bar{x}$	4.04	10.05	40.60	114.0	66.74	6.65	11.25	8.39	61.24	1.676
S	0.036	0.052	0.566	6.231	4.237	0.936	1.595	1.192	2.171	0.005

Natural ageing										
M2Lxy 1	4.18	10.08	42.13	124.8	68.05	7.73	12.63	14.07	55.79	1.416
M2Lxy 2	4.12	10.05	41.41	125.1	68.12	7.69	12.78	13.87	55.47	1.400
M2Lxy 3	4.20	10.01	42.04	124.3	68.34	7.34	12.03	13.98	55.12	1.398
$\bar{x}$	4.17	10.05	41.86	124.7	68.17	7.59	12.48	13.97	55.46	1.405
S	0.042	0.035	0.397	0.404	0.151	0.215	0.397	0.100	0.335	0.010
M2Pxy 1	3.99	10.11	40.34	118.1	70.44	6.82	11.66	13.25	58.36	0.841
M2Pxy 2	4.01	10.01	40.14	117.6	70.45	6.54	11.01	13.01	58.26	0.812
M2Pxy 3	4.00	10.14	40.56	117.9	70.23	6.24	11.12	13.58	58.01	0.856
$\bar{x}$	4.00	10.09	40.35	117.9	70.37	6.53	11.26	13.28	58.21	0.836
S	0.010	0.068	0.210	0.273	0.124	0.289	0.348	0.286	0.180	0.022
M2Pz 1	4.02	10.00	40.20	112.5	66.83	6.67	11.33	10.53	60.11	1.252
M2Pz 2	4.03	10.01	40.34	112.8	66.14	6.54	11.12	10.12	60.14	1.244
M2Pz 3	4.01	10.01	40.14	111.8	66.25	6.23	11.42	10.29	59.87	1.269
$\bar{x}$	4.02	10.01	40.23	112.4	66.41	6.48	11.29	10.31	60.04	1.255
S	0.010	0.006	0.103	0.513	0.371	0.228	0.154	0.206	0.148	0.013
Water absorption 28 days										
M2Lxy 1	4.12	9.98	41.12	106.8	60.52	8.56	14.18	12.57	53.52	1.333
M2Lxy 2	4.19	9.99	41.86	110.2	60.32	8.10	13.20	12.49	55.39	1.257
M2Lxy 3	4.20	9.99	41.96	107.9	58.81	7.80	12.67	12.65	53.31	1.367
$\bar{x}$	4.17	9.99	41.64	108.3	59.88	8.15	13.35	12.57	54.07	1.319
S	0.044	0.006	0.459	1.735	0.935	0.383	0.766	0.080	1.145	0.056
M2Pxy 1	3.99	10.18	40.62	95.6	56.63	7.21	12.33	12.60	49.28	1.293
M2Pxy 2	4.01	10.15	40.70	100.1	58.88	8.01	13.63	12.72	51.58	1.397
M2Pxy 3	4.02	10.16	40.84	97.8	57.21	8.13	13.81	12.44	51.28	1.344
$\bar{x}$	4.01	10.16	40.72	97.8	57.57	7.78	13.26	12.59	50.71	1.345
S	0.015	0.015	0.114	2.250	1.168	0.502	0.808	0.140	1.250	0.052
M2Pz 1	4.04	10.04	40.56	95.6	56.01	8.10	13.69	12.79	49.42	1.281
M2Pz 2	3.99	9.99	39.86	91.1	54.99	7.49	12.81	12.32	48.86	1.353
M2Pz 3	4.05	10.00	40.50	91.1	53.32	7.72	13.01	12.39	46.73	1.297
$\bar{x}$	4.03	10.01	40.31	92.6	54.77	7.77	13.17	12.50	48.34	1.310
S	0.032	0.026	0.388	2.598	1.358	0.310	0.461	0.254	1.419	0.038

Table S8. Flexural properties of 100% recycled material.

No.	h, mm	b, mm	A_0, mm^2	F_{max}, N	σ_{fm}, MPa	ε_{fm}, %	S_{max}, mm	ε_{fp}, %	σ_{fp}, MPa	E_f, GPa
Before exposure										
R2Lxy 1	4.17	10.03	41.83	116.9	64.37	8.44	13.81	12.87	58.81	1.416
R2Lxy 2	4.18	10.20	42.64	118.1	63.62	8.46	13.81	12.70	57.56	1.319
R2Lxy 3	4.13	10.07	41.59	114.7	64.11	7.85	12.98	12.70	56.56	1.243
$\bar{x}$	4.16	10.10	42.02	116.6	64.03	8.25	13.53	12.76	57.64	1.326
S	0.026	0.089	0.549	1.724	0.381	0.343	0.479	0.098	1.127	0.087
R2Pxy 1	3.88	10.14	39.34	88.8	55.88	7.50	13.20	12.82	46.66	1.112
R2Pxy 2	3.96	10.1	40.00	104.6	63.40	7.24	12.47	13.65	49.76	1.455
R2Pxy 3	4.00	10.03	40.12	106.8	63.92	7.49	12.78	12.73	53.81	1.466
$\bar{x}$	3.95	10.09	39.82	100.1	61.07	7.41	12.82	13.07	50.08	1.344
S	0.061	0.056	0.417	9.790	4.499	0.149	0.366	0.507	3.586	0.201
R2Pz 1	4.02	9.98	40.12	106.9	63.63	7.08	12.03	8.60	61.12	1.449
R2Pz 2	4.08	10.01	40.84	119.3	68.70	7.68	12.86	12.00	61.11	1.646
R2Pz 3	4.02	9.97	40.08	108.0	64.35	6.97	11.83	8.55	61.23	1.398

$\bar{x}$	4.04	9.99	40.35	111.4	65.56	7.24	12.24	9.72	61.15	1.498
S	0.035	0.021	0.428	6.864	2.743	0.386	0.546	1.976	0.067	0.131
Ageing in UV chamber 1000 h										
R2Lxy 1	4.02	10.26	41.25	111.3	64.47	7.43	12.62	13.22	52.75	1.430
R2Lxy 2	4.22	10.12	42.71	124.8	66.51	8.67	14.02	13.46	56.92	1.252
R2Lxy 3	4.19	10.18	42.65	123.8	66.47	8.00	13.03	13.06	60.43	1.375
$\bar{x}$	4.14	10.19	42.20	120.0	65.82	8.03	13.22	13.25	56.70	1.352
S	0.108	0.070	0.829	7.522	1.166	0.619	0.720	0.201	3.845	0.091
R2Pxy 1	4.02	10.12	40.68	108.0	63.40	7.54	12.79	12.86	55.47	1.421
R2Pxy 2	4.01	10.12	40.58	109.1	64.36	7.43	12.65	13.63	55.75	0.744
R2Pxy 3	3.98	10.13	40.32	99.0	59.23	7.18	12.31	12.97	49.81	0.537
$\bar{x}$	4.00	10.12	40.53	105.4	62.33	7.38	12.58	13.15	53.68	0.901
S	0.021	0.006	0.188	5.541	2.727	0.183	0.247	0.416	3.352	0.462
R2Pz 1	4.05	9.96	40.34	108.0	63.46	6.64	11.19	8.33	49.01	0.691
R2Pz 2	4.02	9.96	40.04	82.1	48.97	3.96	6.72	6.84	45.00	1.254
R2Pz 3	4.00	9.95	39.80	102.3	61.72	6.40	10.92	7.93	52.50	0.525
$\bar{x}$	4.02	9.96	40.06	97.5	58.05	5.67	9.61	7.70	48.84	0.823
S	0.025	0.006	0.270	13.610	7.911	1.483	2.505	0.772	3.753	0.382
Natural ageing										
R2Lxy 1	4.17	10.10	42.12	122.6	67.01	7.47	12.23	13.27	58.41	1.404
R2Lxy 2	4.20	10.01	42.04	122.1	66.98	7.52	12.01	13.05	58.42	1.412
R2Lxy 3	4.12	10.00	41.20	121.8	67.23	7.34	12.12	13.35	58.34	1.435
$\bar{x}$	4.16	10.04	41.79	122.2	67.07	7.44	12.12	13.22	58.39	1.417
S	0.040	0.055	0.509	0.404	0.137	0.093	0.110	0.155	0.044	0.016
R2Pxy 1	3.89	10.13	39.41	106.8	66.92	7.49	13.14	12.92	57.05	0.767
R2Pxy 2	4.01	10.01	40.14	106.1	66.54	7.12	13.01	12.54	57.10	0.777
R2Pxy 3	3.99	10.09	40.26	107.2	66.12	7.25	13.89	13.01	56.98	0.745
$\bar{x}$	3.96	10.08	39.93	106.7	66.53	7.29	13.35	12.82	57.04	0.763
S	0.064	0.061	0.462	0.557	0.400	0.187	0.475	0.249	0.060	0.016
R2Pz 1	4.03	9.96	40.14	102.3	60.74	6.01	10.18	9.06	24.72	0.613
R2Pz 2	4.01	10.01	40.14	102.0	60.75	5.98	10.01	9.23	24.25	0.599
R2Pz 3	4.01	9.99	40.06	102.6	60.54	5.89	10.23	8.87	24.58	0.602
$\bar{x}$	4.02	9.99	40.11	102.3	60.68	5.96	10.14	9.05	24.52	0.605
S	0.012	0.025	0.046	0.300	0.118	0.063	0.115	0.180	0.241	0.007
Water absorption 28 days										
R2Lxy 1	4.18	10.12	42.30	105.7	57.36	8.31	13.58	12.55	51.28	1.146
R2Lxy 2	4.15	10.11	41.96	97.8	53.92	8.64	14.22	12.30	48.96	1.083
R2Lxy 3	4.24	10.18	43.16	101.2	53.06	9.23	14.86	12.26	48.94	1.054
$\bar{x}$	4.19	10.14	42.47	101.6	54.78	8.73	14.22	12.37	49.73	1.094
S	0.046	0.038	0.621	3.963	2.275	0.466	0.640	0.157	1.345	0.047
R2Pxy 1	4.00	10.08	40.32	85.4	50.86	7.45	12.71	12.21	44.82	1.095
R2Pxy 2	4.03	10.12	40.78	86.6	50.58	7.91	13.40	12.14	45.33	1.271
R2Pxy 3	4.01	10.08	40.42	84.3	49.96	7.98	13.58	12.21	44.63	1.079
$\bar{x}$	4.01	10.09	40.51	85.5	50.47	7.78	13.23	12.19	44.93	1.148
S	0.015	0.023	0.244	1.125	0.461	0.289	0.459	0.040	0.362	0.107
R2Pz 1	4.03	10.01	40.34	89.9	53.12	7.19	12.18	11.23	41.31	1.326
R2Pz 2	4.04	10.03	40.52	83.2	48.79	6.90	11.66	11.62	41.25	1.257
R2Pz 3	3.96	9.95	39.40	71.9	44.27	7.30	12.58	9.77	37.50	1.022
$\bar{x}$	4.01	10.00	40.09	81.7	48.73	7.13	12.14	10.87	40.02	1.202
S	0.044	0.042	0.601	9.093	4.425	0.205	0.461	0.977	2.183	0.159
